# In Situ TEM Multi-Beam Ion Irradiation as a Technique for Elucidating Synergistic Radiation Effects

**DOI:** 10.3390/ma10101148

**Published:** 2017-09-29

**Authors:** Caitlin Anne Taylor, Daniel Charles Bufford, Brittany Rana Muntifering, David Senor, Mackenzie Steckbeck, Justin Davis, Barney Doyle, Daniel Buller, Khalid Mikhiel Hattar

**Affiliations:** 1 Radiation Solid Interactions, Sandia National Laboratories, Albuquerque, NM 87185, USA; ctaylo@sandia.gov (C.A.T.); dcbuffo@sandia.gov (D.C.B.); brmunti@sandia.gov (B.R.M.); mackenzie.steckbeck@ttu.edu (M.S.); davisj@unm.edu (J.D.); bldoyle@sandia.gov (B.D.); dlbulle@sandia.gov (D.B.); 2 Analytical Technologies, Sandia National Laboratories, Albuquerque, NM 87185, USA; 3 Energetics Characterization, Sandia National Laboratories, Albuquerque, NM 87185, USA; 4 Energy and Environment, Pacific Northwest National Laboratory, Richland, WA 99354, USA; David.Senor@pnnl.gov

**Keywords:** ion irradiation, triple beam, in situ TEM, synergistic effects, ion implantation, helium bubble, radiation effects

## Abstract

Materials designed for nuclear reactors undergo microstructural changes resulting from a combination of several environmental factors, including neutron irradiation damage, gas accumulation and elevated temperatures. Typical ion beam irradiation experiments designed for simulating a neutron irradiation environment involve irradiating the sample with a single ion beam and subsequent characterization of the resulting microstructure, often by transmission electron microscopy (TEM). This method does not allow for examination of microstructural effects due to simultaneous gas accumulation and displacement cascade damage, which occurs in a reactor. Sandia’s in situ ion irradiation TEM (I^3^TEM) offers the unique ability to observe microstructural changes due to irradiation damage caused by concurrent multi-beam ion irradiation in real time. This allows for time-dependent microstructure analysis. A plethora of additional in situ stages can be coupled with these experiments, e.g., for more accurately simulating defect kinetics at elevated reactor temperatures. This work outlines experiments showing synergistic effects in Au using in situ ion irradiation with various combinations of helium, deuterium and Au ions, as well as some initial work on materials utilized in tritium-producing burnable absorber rods (TPBARs): zirconium alloys and LiAlO_2_.

## 1. Introduction

The materials design process for advanced nuclear fission and fusion reactors requires understanding material behavior in extreme environments. Structural reactor materials must satisfy standard materials design criteria based on mechanical properties, thermal creep, cyclic fatigue and combined creep-fatigue, as well as adequate resistance to radiation damage and chemical degradation (e.g., corrosion and stress corrosion cracking) [1]. Material response to radiation damage is typically investigated by either placing samples in a test reactor, or by utilizing ion irradiation to simulate neutron damage. Ion irradiation offers the advantage of more rapid damage accumulation (which often reduces experimental times from months or years to hours or days) and often does not leave samples with residual radioactivity. Most ion accelerator facilities offer the capability of irradiating with a range of ion species and energies that simulate the defect cascades produced by neutron irradiation, or implanting with lower energy gas ions to simulate bubble formation. These experiments neglect the concurrent effect of defect-gas atom interaction that occurs in a real reactor irradiation environment. Some facilities, e.g., the Joint Accelerators for Nano-science and Nuclear Simulation (JANNUS) [2] and the Takasaki Ion Accelerators for Advanced Radiation Application (TIARA) [3], have developed multi-beam irradiation capabilities, which allow for the exploration of these synergistic effects. The combined effects can be striking; for instance, Tanaka et al. demonstrated minimal swelling in Fe-Cr alloys when irradiated with concurrent Fe/He and Fe/H beams, but more than an order of magnitude increase in swelling and void formation during irradiation with all three species at once [4]. While clearly important, experiments that elucidate these effects remain difficult to realize in practice, in terms of both the necessary irradiation and microstructural characterization capabilities.

Early electron microscopists identified in situ TEM as a tool that would elucidate the role of crystal defects, give accurate kinetic measurements of reaction rates and provide evidence for the mechanisms of dynamic processes through direct observation [5]. Electron beam irradiation effects quickly became one of the first topics studied within in situ TEM due to the electron beam damage innately experienced by materials inside the microscope once the knock-on energy is exceeded. Depending on the threshold displacement energy of atoms inside the material, even a 200 keV electron beam can produce Frenkel pair damage in some materials. High voltage electron microscopes facilitated the creation of point defects and the subsequent direct observation of their effects. Ion irradiation performed in situ in the TEM allowed for direct observation of more complicated radiation effects. In situ ion irradiation in the TEM quickly became an important technique and has been thoroughly reviewed elsewhere [6,7,8,9]. The first in situ ion irradiations were performed unintentionally in 1961 due to the emission of O^−^ ions from a contaminated tungsten electron gun filament [10] and were initially employed intentionally in 1966 by using O^−^ ion beams generated by BaCo_3_- or SrCo_3_-coated filaments inside the electron microscope [11]. Researchers at Harwell [12] first generated a heavy-ion beam by connecting an ion accelerator to an electron microscope. Similar facilities were created shortly after by researchers at the University of Virginia, University of Tokyo, Argonne National Laboratory, and many others.

Today, the in situ ion irradiation TEM (I^3^TEM) [13] at Sandia National Laboratories combines single, double and triple ion beam irradiation with the real-time visualization power of in situ TEM. The ability to irradiate with two or three ion beams simultaneously allows for exploration of synergistic effects between He gas accumulation, cascade damage, H isotopes and temperature, making the I^3^TEM a unique facility for investigating radiation effects in materials. This work presents initial results of in situ triple ion irradiation experiments on a variety of materials, beginning with the discussion of the accelerator beam line optics required to guide three ions of a vastly different mass energy product into the TEM through a single port, then discussing triple ion beam irradiations in a model face-centered cubic (FCC) system, Au, followed by initial results of triple ion irradiation in materials utilized in tritium-producing burnable absorber rods (TPBARs), zirconium alloys and LiAlO_2_.

## 2. Materials and Methods 

### 2.1. Accelerator Beam Line Design

**Figure 1:**
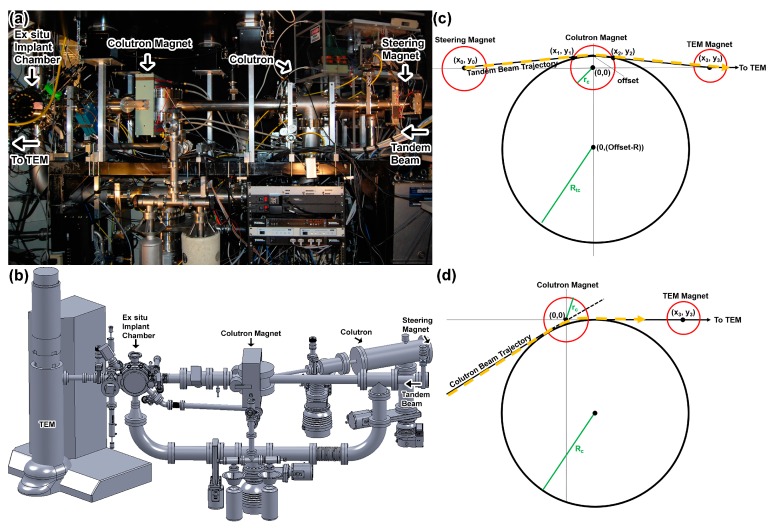
Diagrams showing the I^3^TEM facility, starting with (**a**) an image of the beam line, marked with arrows to show the Colutron magnet, Tandem electrostatic steerers and the original locations of the Colutron and Tandem beams; (**b**) a Solid Works drawing of the beam line in (**a**), clearly showing the Colutron and Tandem beam lines joining at the Colutron magnet; (**c**) the Tandem beam trajectory through the Colutron magnet; and (**d**) the Colutron beam trajectory through the Colutron magnet, indicated clearly with dashed yellow lines.

The I^3^TEM facility, discussed in detail in Hattar et al. [13], consists of a JEOL 2100(HT) LaB_6_ microscope (JEOL USA, Peabody, MA, USA), connected to an accelerator beam line capable of receiving beams from two accelerators: a 10-kV Colutron G-1 ion accelerator (Colutron Research Corporation, Longmont, CO, USA) and a 6-MV Van de Graaff-Pelletron Tandem Accelerator (High Voltage Engineering Corporation, Burlington, MA, USA). Ion beams from the Colutron are accelerated to a maximum energy of 10q keV, where q is the ion charge state, and bent 20° toward the microscope with an electromagnet. The ion beam from the Tandem enters the beam line along the TEM axis (Figure 1). Most typical Colutron beams are keV energy ^4^He^+^ and ^2^D_2_^+^. These beams simulate the effects of neutron-induced transmutation through (n,α) and (n,p) nuclear reactions in materials used in nuclear reactors. Deuterium ions were selected instead of protium to match the mass energy product of ^4^He^+^ and to ease identification in post-mortem chemical analysis. By matching the beam rigidity, both beams are steered identically by the Colutron bending magnet and TEM lenses, permitting both ^4^He^+^ and ^2^D_2_^+^ to be produced by the same Colutron and bent into the TEM simultaneously.

The bending electromagnet of the Colutron slightly deflects the Tandem beam, in some cases causing the beam to miss the TEM sample. To alleviate this problem, steering magnets installed before the Colutron bending magnet are used to offset the trajectory of the Tandem beam from the axis of the Colutron magnet and ultimately to deflect the ions in the direction of the TEM target. In addition to these two magnets, there is a strong, constant magnetic field around the samples produced by the objective lenses inside the TEM, which affects the trajectory of the light, low energy Colutron beam. The Colutron beam must therefore be offset from the axis of the TEM such that the circular trajectory of ions in this magnetic field directs them to the center of the sample. Figure 1c shows the trajectory of ion beams produced by the Tandem accelerator through the Tandem steering magnets and the Colutron magnet, and Figure 1d shows the trajectory of ion beams produced by the Colutron through the Colutron magnet.

### 2.2. Characterization of the He + D Beam 

**Figure 2:**
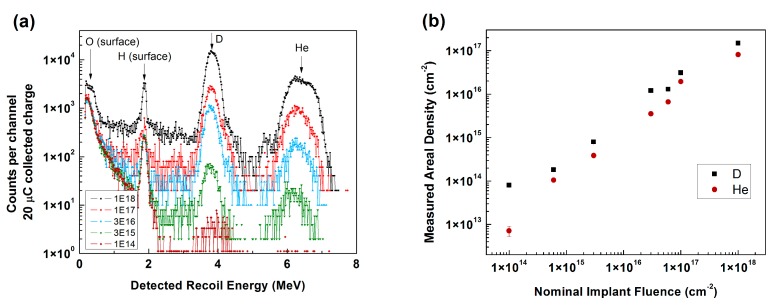
Elastic recoil detection (ERD) results from He + D implantations into Si wafers, showing labeled peaks for select fluences in (**a**) and the measured areal density as a function of ion implantation fluence in (**b**). Most error bars are smaller than the data points in (**b**). Note that the scales are log (vertical) in (**a**) and log-log in (**b**).

Experiments were performed to quantify the He/D ratio coming from the single Colutron ion beam. Beam current was estimated during experiments from a Faraday cup biased to 300 V to suppress electron emission. Implantations of 1 × 10^14^, 6 × 10^14^, 3 × 10^15^, 3 × 10^16^, 6 × 10^16^, 1 × 10^17^ and 1 × 10^18^ ions/cm^2^ were performed into silicon wafers using the ex situ implantation chamber seen in Figure 1a,b to investigate the composition of the He + D beam from the Colutron. Elastic recoil detection (ERD) [14] experiments were performed with a 24 MeV Si^4+^ probe beam. The sample was tilted 15° towards the detector, and a 30° recoil angle was utilized. A 13.5-m Mylar range foil was positioned in front of the detector to screen incoming Si recoils. A known standard of SiN implanted with H was utilized for quantification purposes. Results from the ERD experiments appear in Figure 2. For clarity, the 6 × 10^14^ and 6 × 10^16^ ions/cm^2^ samples have been omitted from Panel (a). Peaks arising from O and H present at the sample surfaces were identified consistently, and peaks for the D and He indicate that both species reached the sample in the measured area. Quantification of the results revealed that concentrations were approximately one order of magnitude lower than the nominal fluences estimated from the Faraday cup measurement and that the ratio of D to He remained around 1.8.

### 2.3. Experimental Details

A variety of TEM stages are available for use at the Sandia I^3^TEM facility, enabling studies of synergy between ion irradiation damage and environmental effects. The angle of tilt (around +30° in *x*) required for the ion beam to hit the sample inside each holder has been previously determined [13]. Ion beams are aligned onto the sample stage with a piece of quartz attached to a JEOL single-tilt stage, as described in Hattar et al. [13], with each beam aligned individually in the case of double and triple ion irradiation experiments. Ion and electron beam spots are burned into tape to confirm their overlap prior to starting the experiment. The beam area used to calculate the total fluence is estimated from the burn spot area. In this work, room temperature experiments were performed using the JEOL single tilt stage, and high temperature experiments were performed using the Hummingbird 2.3-mm heating stage. TEM images were analyzed using ImageJ [15].

The energy of ions produced by the Colutron is typically <20 keV, so that they are implanted within the TEM foil thickness. The ^2^D_2_^+^ molecule breaks apart instantaneously upon hitting the sample, becoming 50% of its initial energy. In the case of a 10 keV ^2^D_2_^+^ ion beam, 5 keV D will be implanted into the sample. Ions produced by the Tandem accelerator are typically in the MeV energy range and of much higher mass (e.g., ^197^Au) and result in high levels of damage similar to that experienced by neutron-irradiated materials in reactors, but in a much shorter timeframe. Irradiation parameters were determined using Quick Calculations in the Monte Carlo-based Stopping and Range of Ions in Matter (SRIM) code [16]. SRIM calculations are performed at an angle of 60° off surface normal because the sample holder is tilted 30° toward the beam line, which is normal to the TEM. A threshold displacement energy, *E_D_*, of 43 eV was used for Au [17].

**Figure 3:**
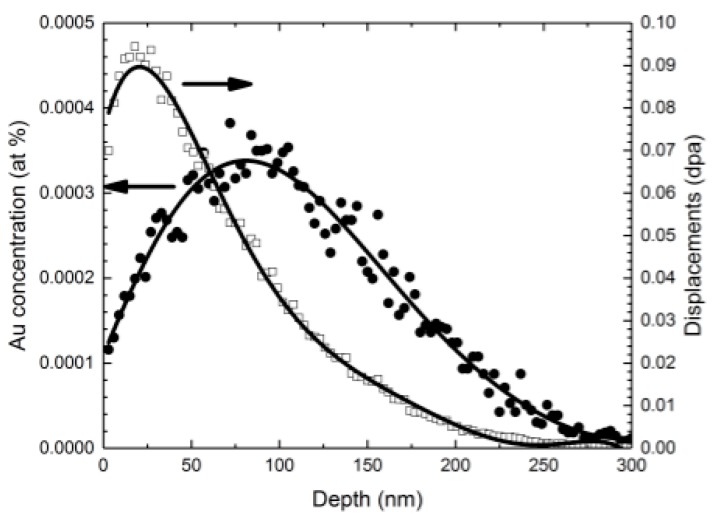
Stopping and Range of Ions in Matter (SRIM) predicted Au concentration (at %) and damage dose (dpa) for 2.8 MeV Au^4+^ into a Au foil for a fluence of 3.6 × 10^12^ ions/cm^2^. Filled circles indicate the Au concentration profile, and empty squares indicate the Au displacement damage profile. Lines of fit are only meant to guide the eye.

Au TEM samples were prepared by pulsed-laser deposition (PLD) depositing of 40 nm-thick Au films onto NaCl single crystals, which were cleaved and dissolved in water, leaving the Au foil to be floated off onto a TEM grid. TEM samples were subsequently annealed to increase the grain size. An initial experiment was performed using two different ion fluxes of 2.8 MeV Au^4+^. In the high flux experiment, an ion beam current of 6.2 nA (9.69 × 10^10^ ions/cm^2^·s) was used, and the sample was irradiated in situ for 38 s. In the low flux experiment, 60 pA (9.38 × 10^8^ ions/cm^2^·s) was used, and the sample was irradiated for 3800 s. Both samples were irradiated to a fluence of 3.6 × 10^12^ ions/cm^2^. The area of the 2.8 MeV Au^4+^ beam spot was estimated to be 10 mm^2^. Figure 3 shows SRIM calculations of displacement damage (dpa) and the implanted ion concentration for 2.8 MeV Au^4+^ into a Au foil.

**Figure 4:**
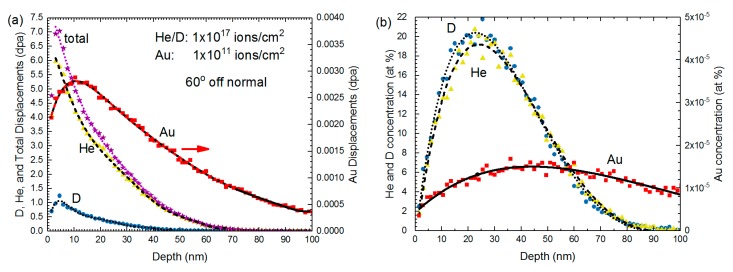
SRIM calculated (**a**) displacement and (**b**) concentration profiles as a function of depth for Au irradiated with various ion species, including 10 keV He, 5 keV D and 1.7 MeV self-ions. He and D ion fluence of 1 × 10^17^ ions/cm^2^ and Au fluence of 1 × 10^11^ ions/cm^2^ were used in the calculations. SRIM calculations are done at 60° off surface normal because the TEM specimen holder is tilted toward the ion beam. The total dpa profile (D + He + Au) is indicated by purple stars in (**a**); the Au profiles are indicated by red squares; He profiles are indicated by yellow triangles; and D profiles are indicated by blue circles. Lines of fit are only meant to guide the eye.

Next, a series of samples were irradiated in situ at room temperature with various combinations of 10 keV He, 5 keV D (resulting from a 10 keV D_2_ beam) and 1.7 MeV Au^3+^. SRIM calculations for these beams into a Au foil are shown in Figure 4. Note that, in the case of He and D implantations, most of the gas will readily diffuse to the surface of the thin film. The actual implanted gas concentration is expected to be significantly lower than the SRIM calculated values. Single-ion irradiations include: He, D and Au; and double ion irradiations include: He + D, D + Au and He + Au. A final triple ion irradiation was done using all three beams simultaneously. He and He/D beam spot areas were estimated to be 19 mm^2^ for all calculations, based on multiple beam spot area measurements. All ion beam fluxes were held relatively constant, with He^+^ current ranging from 1.2 to 2.6 μA (3.95 × 10^13^–8.55 × 10^13^ ions/cm^2^·s) and the Au^3+^ current ranging from 5 to 20 pA (1.04 × 10^8^–4.17 × 10^8^ ions/cm^2^·s, or ≈2.9 × 10^−6^ dpa/s) between the experiments. Higher Au currents resulted in samples quickly becoming saturated with damage, making comparisons between various irradiation conditions difficult. All samples were irradiated for at least 30 min. When calculating fluences, the combined He/D_2_ beam was estimated to be 50% He and 50% D_2_ based on the ERD results presented above, where the D/He ratio was determined to be 1.8, because measuring the exact He/D_2_ ratio before each experiment would be impractical. The He fluence was therefore taken as 50% of the total fluence, calculated using the current recorded by the Faraday cup, and the D fluence was taken as 2 × 50% of the total fluence because the Faraday cup registers one count per D_2_ molecule. Specific damage dose and implanted gas concentrations are estimated within the text. Based on SRIM calculations, the Au ions produce about two orders of magnitude more displacements than the D and He beams. Displacement damage produced by Au ions results in displacement cascades, similar to neutron irradiation damage, as opposed to the point defect damage expected to result from implantation with low energy, low mass D and He beams. Much of the 1.7 MeV Au beam travels through a 100 nm-thick TEM foil of most materials, while all of the He and D will implant directly into the TEM foil, providing a mechanism for studying gas bubble formation in situ.

Preliminary experiments were performed on zirconium alloys and LiAlO_2_ with the goal of understanding synergistic effects taking place in TPBAR materials inside a reactor. An understanding of defect-gas interactions and bubble formation is of current interest in these materials. All zirconium alloy TEM samples were prepared by jet polishing. In these experiments, various zirconium alloys (primarily Zircaloy-4 and optimized ZIRLO) were irradiated with combinations of 10 keV He, 5 keV D and 3 MeV Zr in situ at 310 °C. In both this and the LiAlO_2_ experiments, elevated temperature was used to simulate reactor conditions. Here, we present results from the He, He + D and triple ion irradiation experiment with all three ions simultaneously. In these preliminary experiments, the zirconium alloy varied, and the ion beams were left overnight in many instances, so the exact ion flux and total ion fluence are unknown.

All LiAlO_2_ samples were prepared from LiAlO_2_ powder drop-cast onto 2.3-mm Mo grids with a C film. LiAlO_2_ samples were irradiated in situ at 310 °C using various combinations of 10 keV He, 5 keV D and 1.7 MeV Au. SRIM calculations were done using *E_D_* = 10 eV for Li, 30 eV for O and 27 eV for Al [18]. In this manuscript, we present initial results from the He (single ion), He + D (double ion) and He + D + Au (triple ion) irradiation experiments. The 10 keV He + D_2_ beam was tuned in the morning and used for both the double and triple ion experiments, so that conditions remained as uniform as possible. In the single ion (10 keV He) experiment, the He beam current was 4 μA (ion flux of 1.87 × 10^14^ ions/cm^2^·s), possibly lower based on the burn spot, which indicated that the edge of the He beam profile was hitting the observable area of the sample. The sample was irradiated for 2 h, a total fluence of 1.34 × 10^18^ ions/cm^2^. Again, note that, due to the particles being 50–100 nm thick, most of the He is expected to diffuse to the surface rather than implanting inside the sample, and the actual amount remaining inside the sample should be much lower than the SRIM predicted value. The sample was exposed to the electron beam for most of the implantation. In the double ion experiment (10 keV He + D_2_), the beam current was 3 μA (ion flux of 9.38 × 10^13^ ions/cm^2^·s). The sample was irradiated for 2 h in situ, resulting in a total fluence of 6.75 × 10^17^ (He + D_2_)/cm^2^. In this experiment, the sample was imaged every 5 min, exposed for ≈30 s each time, plus a few minutes total for re-alignment purposes. For the remainder of the irradiation, the electron beam was off. In the triple ion irradiation experiment, the He/D_2_ beam parameters were kept exactly the same as the dual beam experiment. The Au^3+^ particle current was ≈1.08 nA (7.18 × 10^10^ ions/cm^2^·s) on average during this experiment. The total irradiation time was 2 h, resulting in the same He/D fluences as the double beam experiment and a Au fluence of 5.17 × 10^14^ ions/cm^2^. The combined peak damage dose for the triple ion experiment was 42 dpa. The sample was exposed to the electron beam for the entirety of the single and triple ion irradiation experiments.

## 3. Results

### 3.1. Dose Rate Effects of In Situ Single Beam Au Irradiation into Au

**Figure 5:**
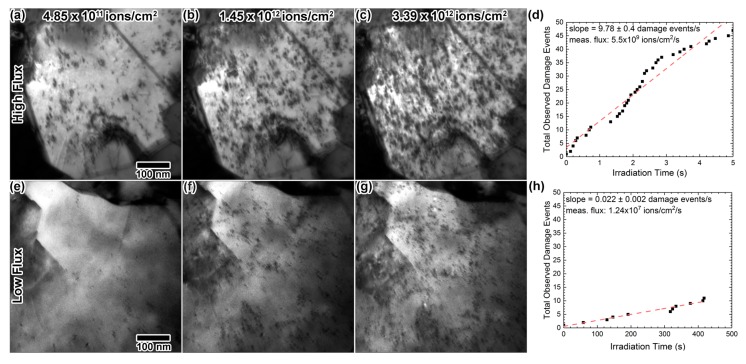
TEM images from in situ 2.8 MeV Au^4+^ irradiation into a Au foil using dose rates of 9.69 × 10^10^ (**a**–**c**) and 9.38 × 10^8^ ions/cm^2^·s (**e**–**g**), at fluences of 4.85 × 10^8^, 1.45 × 10^12^ and 3.39 × 10^12^ ions/cm^2^. (**d**,**h**) Plots of the number of observable damage events as a function of time, for the first 5 s of the high flux experiment and for the first 500 s of the low flux experiment, fit with a linear curve indicated by the pink dashed line. The slope of the linear fit was used to determine the measured flux. The entire irradiation time is not included in (**d**,**h**) because new damage events become difficult to distinguish from existing damage once the microstructure nears damage saturation. All TEM images were taken at the same magnification.

First, Au foils nominally 40 nm in thickness were irradiated in situ with 2.8 MeV Au^4+^ to the same total fluence, 3.6 × 10^12^ ions/cm^2^, using two different fluxes of 9.38 × 10^8^ and 9.69 × 10^10^ ions/cm^2^·s. This equates to 0.09 displacements per atom (dpa) and peak damage dose rates of 2.5 × 10^−5^ and 2.5 × 10^−3^ dpa/s. Though not ideal, samples were not aligned to a specific imaging condition due to the relatively small grain size. An attempt was made at maintaining a similar imaging condition for all samples throughout all in situ experiments by examining the defect and grain contrast. Figure 5a–c shows excerpts of the high flux irradiation, and Figure 5e–g shows excerpts of the low flux irradiation at the same fluence. The images in Figure 5e–g taken from the low flux experiment have less observable damage than the images in Figure 5a–c from the high flux experiment. The number of observed damage events per unit time was expectedly higher with a higher ion flux. In the high flux experiment, a multitude of defects was observed after only 5 s of irradiation, at 4.85 × 10^11^ ions/cm^2^, where only a few defects were observed in the low flux experiment. With high flux, the defect concentration increased steadily for ≈30 s, until defect saturation seemed to occur. A high density of irradiation-induced defects obscured most other microstructural features after 3.39 × 10^12^ ions/cm^2^. In the low flux experiment, the defect concentration continued to increase throughout the irradiation, reaching a defect concentration appearing to be lower than that observed in the high dose rate experiment. Additionally, defect clusters generally appeared to be smaller in size than those produced in the high rate experiment. 

Figure 5d,h shows the number of observable damage events as a function of time, during the first 5 s of the high flux irradiation and during the first 417 s of the low flux irradiation, taken from the in situ images. Note that this is only a portion of each experimental time; the entire series of images was not used because once the sample accumulates enough defects, new damage events became difficult to distinguish. New damage events became difficult to distinguish after about 5 s in the high flux data. Thus, the plot in Figure 5d extends to 5 s, and the damage events were recorded for about two orders of magnitude longer for the low flux data (approximately the same fluence). The ion flux should, however, remain constant for the entire irradiation. Assuming a constant ion flux, the slopes of the linear fits of these plots were utilized to extract a mean ion flux from the in situ videos. Calculated ion fluxes were 1.24 × 10^7^ and 5.5 × 10^9^ ions/cm^2^·s for the low and high dose rate experiments, respectively, both lower than the actual flux. This is likely a combination of (i) only a portion of the damage events being observable in the imaging conditions show in in Figure 5 and (ii) many ions losing energy purely due to electronic stopping as they pass through the 40 nm-thick foil. SRIM calculations (Figure 3) indicate that most Au ions will pass directly through the Au foil during these experiments. 

### 3.2. In Situ Single Ion Irradiation into Au

**Figure 6:**
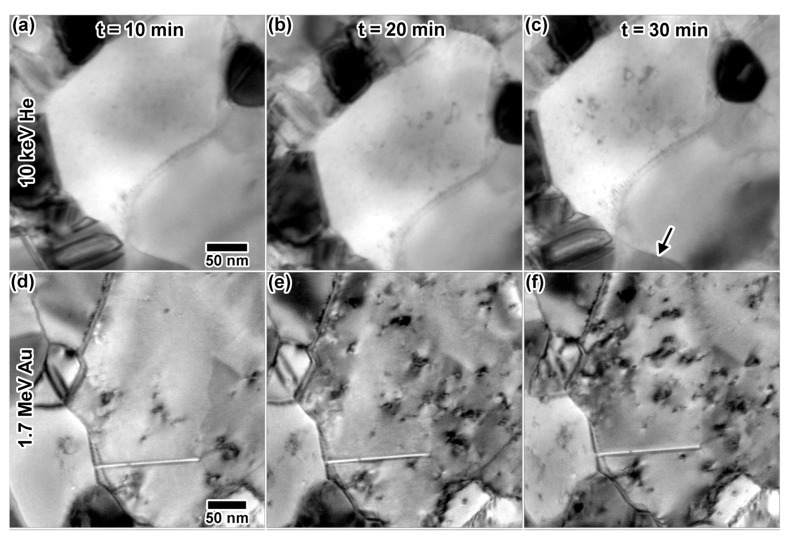

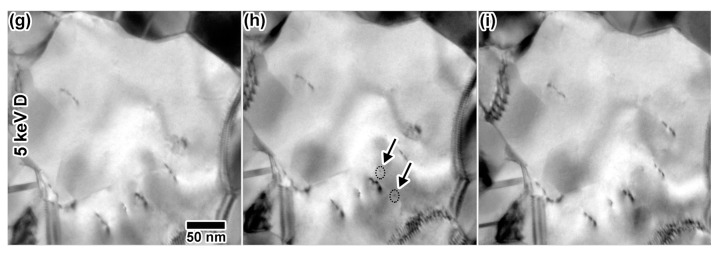
In situ TEM images from single beam irradiations: (**a**–**c**) 10 keV He; (**d**–**f**) 1.7 MeV Au and (**g**–**i**) 5 keV D after 10, 20 and 30 min. The arrow in (**c**) is pointing toward a grain where bubbles are easily visible (shown zoomed in Figure 7). Dashed circles in (**h**) highlight defects that were observed and later disappeared. At 30 min, irradiation doses were as follows: (**c**) 1.54 × 10^17^ ions/cm^2^, 9.2 dpa, 31.97 at % He, (**f**) 7.5 × 10^11^ ions/cm^2^, 0.02 dpa, and (**i**) 1.42 × 10^17^ ions/cm^2^, 1.8 dpa, 30.94 at % D. All TEM images were taken at the same magnification.

**Figure 7:**
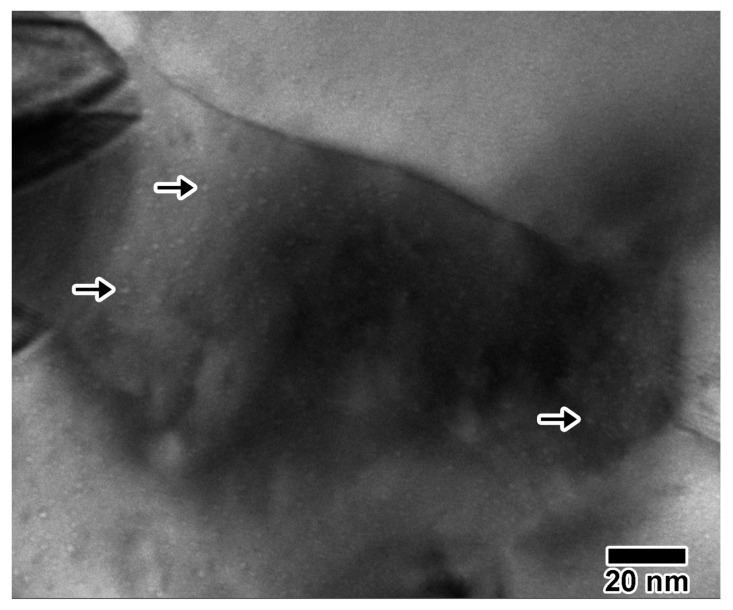
Still image taken after single ion 10 keV He implantation into Au, showing a 1.5-nm bubble distribution in underfocus. This is a zoomed image of the grain indicated by an arrow in Figure 6c. A few bubbles are indicated by arrows.

Figure 6 shows (a–c) excerpts of the 10 keV He irradiation; (d–f) excerpts of the 1.7 MeV Au irradiation and (g–i) excerpts of the 5 keV D irradiation. Within 20 min of irradiation with 10 keV He, a few defects were visible, which grew into larger, more complex defect structures after 30 min. Those structures included triangular defects, which may be stacking fault tetrahedra (SFTs). Though bubbles were not observed in the video collected in situ due to sample motion, a still image recorded post-irradiation using a higher resolution camera (Figure 7) indicated a homogenous distribution of circular bubbles ≈1.5 nm in diameter. Bubbles were much more visible in grains with less defect contrast, with an example indicated by an arrow in Figure 6. In situ irradiation with 1.7 MeV Au^3+^ resulted in a microstructure similar to that observed in Figure 5, with the defect concentration increasing with time. Note that, even though the irradiation only reached 0.02 dpa after 30 min and the He implantation reached 9.2 dpa (1.54 × 10^17^ ions/cm^2^) after 30 min, there is much more visible damage in the Au irradiated sample. This illustrates the difference in resulting microstructures from cascade (Au) vs. point defect (He) damage. No cavities were observed. In situ implantation with 5 keV D resulted in very few microstructural changes within 30 min (1.42 × 10^17^ ions/cm^2^, 1.8 dpa). Two defect clusters due to D implantation are shown in Figure 6h, but disappeared and are not visible in Figure 6i.

### 3.3. In Situ Double Ion Irradiation into Au

**Figure 8:**
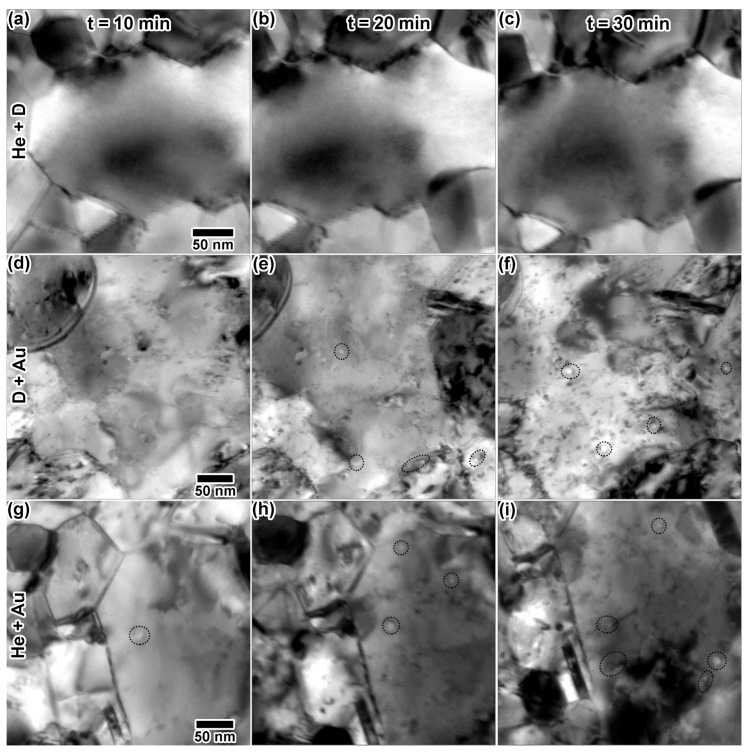
In situ TEM images from double beam irradiations: (**a**–**c**) 10 keV He + 5 keV D; (**d**–**f**) 1.7 MeV Au + 5 keV D and (**g**–**i**) 1.7 MeV Au + 10 keV He after 10, 20 and 30 min. Dashed circles highlight possible cavity formation. At 30 min, irradiation doses were as follows: (**c**) 8.88 × 10^16^ D/cm^2^ and 4.44 × 10^16^ He/cm^2^, 1.1 D dpa and 2.7 He dpa, 19.35 at % D and 9.22 at % He, (**f**) 1.42 × 10^17^ D/cm^2^ and 1.88 × 10^11^ Au/cm^2^, 1.8 D dpa and 0.005 Au dpa, 30.94 at % D, and (**i**) 1.54 × 10^17^ He/cm^2^ and 7.5 × 10^11^ Au/cm^2^, 9.2 He dpa and 0.02 Au dpa, 31.97 at % He. All TEM images were taken at the same magnification.

Figure 8 shows (a–c) excerpts from the He + D irradiation; (d–f) excerpts from the D + Au irradiation and (g–i) excerpts from the He + Au irradiation. In these experiments, the ion fluxes were kept approximately equal to the single ion experiments just described. Dual ion He + D irradiation resulted in very few microstructural changes after 30 min. Some small defects were observed at the end of the irradiation in Figure 8c. Dual ion D + Au irradiation resulted in defect and cavity production. Through-focus imaging [19] was found difficult, especially in situ, due to sample motion and the high levels of defect contrast, but some defects appear to be cavities. It is important to note that distinguishing between large cavities and craters has been proven difficult in Au [20,21]. Cavity-like structures were measured to be 8–12 nm in diameter in Figure 8e and grew as the irradiation continued, measuring at 10–15 nm in diameter in Figure 8f. Cavity size and density differed significantly from those produced by He alone. There was significantly more cavity formation in the Au + D case than in the pure Au or pure D case. 

**Figure 9:**
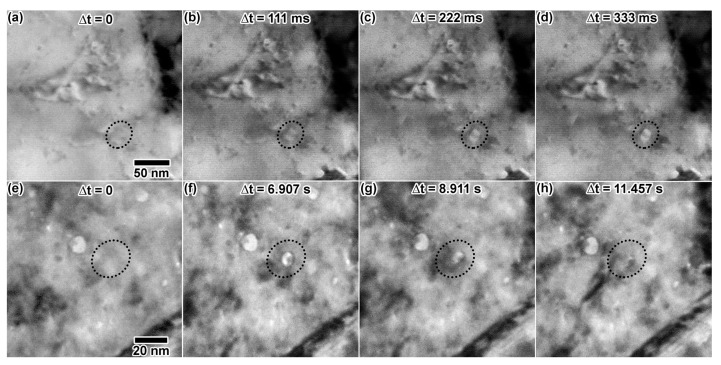
In situ TEM images showing cavity growth as a function of time due to (**a**–**d**) double ion irradiation with 5 keV D + 1.7 MeV Au and cavity formation and collapse as a function of time due to (**e**–**h**) triple ion irradiation with 10 keV He, 5 keV D and 2.8 MeV Au. Dashed circles highlight the cavity of interest in each image. Approximate fluences in (**e**–**h**) were Au: 1.2 × 10^13^, He: 1.3 × 10^15^ and D: 2.2 × 10^15^ ions/cm^2^. TEM images (**a**–**d**) were taken at the same magnification (scale bar in (**a**)), and (**e**–**h**) were taken at the same magnification (scale bar in (**e**)).

Dual ion He + Au irradiation resulted in defects and large cavity-like features. Defect and apparent cavity concentration appeared to increase with damage dose and He concentration. The cavity-like features, indicated by red circles, ranged in size from 7–20 nm throughout the irradiation and tended to exhibit oblong shapes. These features again differed from those observed during the single beam He implantation, where bubbles were consistently smaller and round. Rapid nucleation of cavities was captured in a few instances during multiple ion irradiation. Figure 9a–d shows cavity nucleation during the Au + D irradiation, with the initial microstructure in Figure 9a and the final cavity appearance in Figure 9d. The cavity is first apparent in Figure 9b, although it appears faint, as it nucleated during the acquisition of this video frame. Given the frame rate of 15 FPS, a maximum nucleation time of 111 ms can be established. The cavity appears more clearly in Figure 9c and may have expanded slightly before the stable structure in (d) was reached.

### 3.4. In Situ Triple Ion Irradiation into Au 

**Figure 10:**
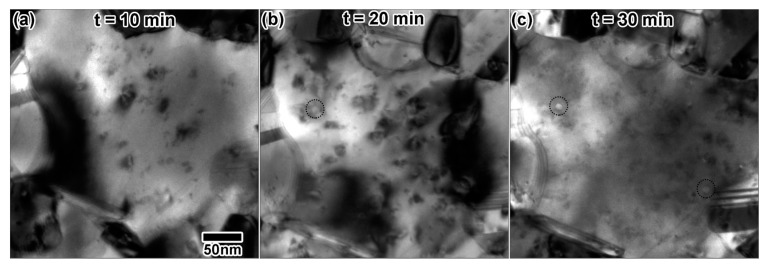
In situ TEM images from triple ion beam irradiation with 10 keV He, 5 keV D and 1.7 MeV Au shown after (**a**) 10 min, (**b**) 20 min and (**c**) 30 min. Dashed circles indicate possible cavity formation. At 30 min, irradiation doses were as follows: 7.11 × 10^16^ D/cm^2^ + 3.55 × 10^16^ He/cm^2^ + 1.88 × 10^11^ Au/cm^2^, 2.1 He + 0.88 D + 0.005 Au dpa and 7.37 at % He + 15.49 at % D. All TEM images were taken at the same magnification.

Figure 10 shows the microstructural progression of Au under triple ion irradiation. Triple ion irradiation resulted in a steady increase in the concentration of defect clusters. An increasing number of structures thought to be large cavities gradually appeared during the irradiation, with one possible cavity at 20 min (Figure 10b), having a measured diameter of 19 nm. After 30 min of irradiation (Figure 10c), more possible cavities are apparent, which had measured diameters of 19–20 nm. Cavities are spread heterogeneously throughout the sample. Some cavities were observed to rapidly appear in a process similar to what was presented in Figure 9a–d. One instance is illustrated in Figure 9e–h, where a cavity both nucleated and collapsed quickly during a separate triple ion irradiation experiment using 2.8 MeV Au, 10 keV He and 5 keV D. The microstructure prior to cavity nucleation is shown in Figure 9e. Within a few frames, the fully-grown cavity is shown in Figure 9f. Defect clusters caused by incoming ions appeared in the vicinity of the cavity, and the cavity began to disappear in Figure 9g. After a few seconds, the cavity is no longer visible in Figure 9h.

### 3.5. In Situ Single, Double and Triple Ion Irradiation in TPBAR Materials

Tritium-producing burnable absorber rods (TPBARs) consist of a lithium aluminate (LiAlO_2_) pellet that is surrounded by inner and outer layers of Zircaloy-4. The rods are designed for placement inside a nuclear reactor, currently the TVA Watts Bar reactor. The outer layer acts as a getter for ^3^H, and the inner layer acts to reduce tritium oxide (^3^H_2_O) emerging from the pellet so that ^3^H can be gettered. The LiAlO_2_ pellet is enriched with the ^6^Li isotope, which absorbs neutrons from the reactor, becoming ^7^Li, which fissions to ^3^H + ^4^He in less than 1 s. Tritium will eventually β-decay to ^3^He with a half-life of 12.3 years. TPBARs are surrounded by reactor-grade stainless steel cladding with an aluminide coating to prevent inward diffusion of hydrogen from the coolant and outward diffusion of tritium [22]. Predicting the longevity of the TPBAR relies on a thorough understanding of gas-defect interactions inside both the LiAlO_2_ pellet and the Zircaloy-4 at elevated temperatures. The damage dose of pellets inside the Watts Bar reactor is expected to be approximately 20 dpa.

**Figure 11:**
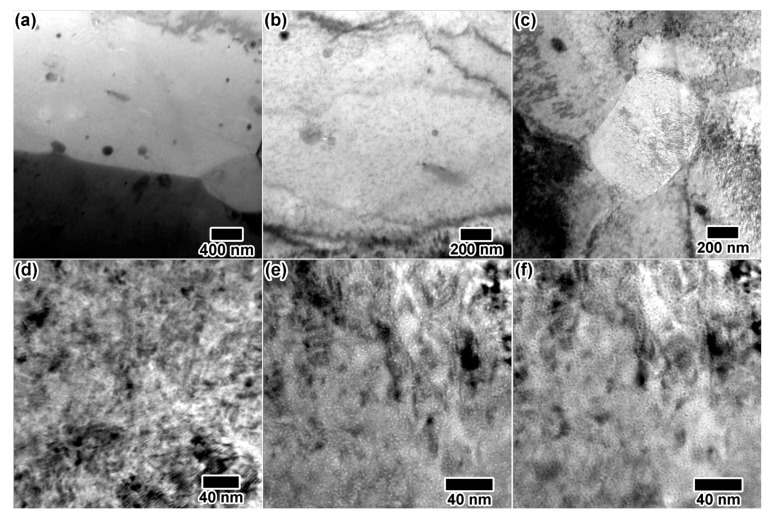
Pristine zirconium alloy structure is shown in (**a**). In situ TEM images from triple ion beam irradiation of various zirconium alloys with (**b**) 10 keV He, (**c**) 10 keV He + 3 MeV Zr and (**d**) 10 keV He + 5 keV D + 3 MeV Zr. Cavities observed 30 days after irradiation are shown in (**e**) underfocus and (**f**) overfocus.

Figure 11a shows the initial microstructure of zirconium alloy (ZIRLO and Zircaloy-4) samples. Black, circular regions shown in Figure 11a are possibly precipitates. Zirconium alloys were irradiated at 310 °C with various combinations of 10 keV He, 5 keV D and 3 MeV Zr. A high defect density was produced by 10 keV He irradiation (Figure 11b), but no bubble formation was observed. Dual ion He + Zr irradiation (Figure 11c) resulted in a higher density of defects than the single ion irradiation, and triple ion irradiation (Figure 11d) resulted in a microstructure completely saturated with defects. The high levels of defect contrast in the double and triple ion irradiations made cavity observation and defect analysis difficult. No cavities were observed in any of the irradiated samples during the in situ experiments. Samples were stored for 30 days at room temperature in vacuum after the experiment, then characterized. After 30 days, a high density of homogenously-distributed circular cavities was observed in all He implanted samples, an example of which is shown in Figure 11e–f.

**Figure 12:**
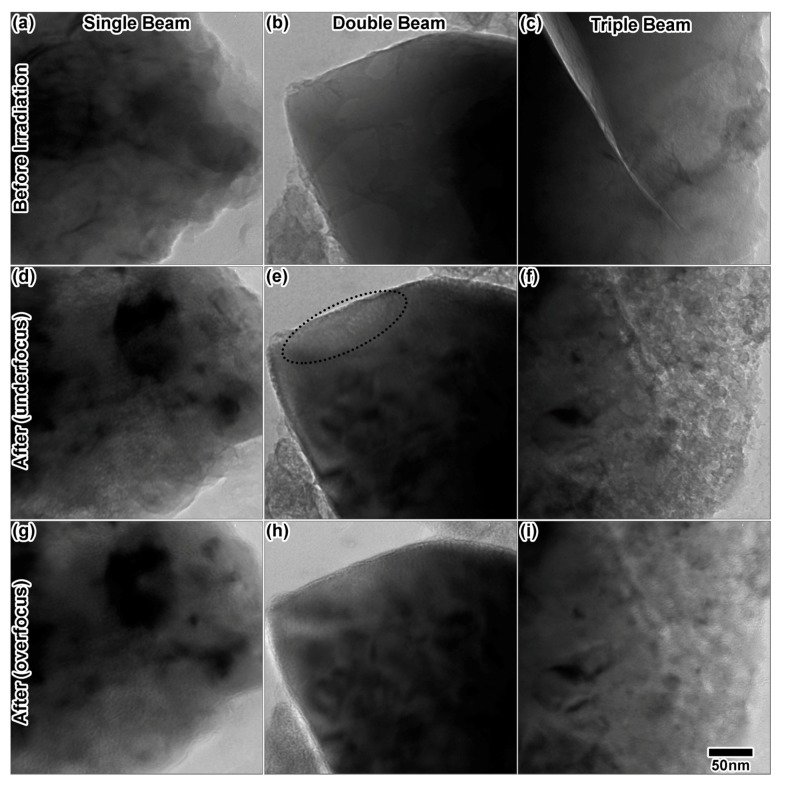
TEM images from before and after single, double and triple ion irradiation at 310 °C in LiAlO_2_. (**a**–**c**) The particles before irradiation; (**d**–**f**) The particles in underfocus after irradiation and (**g**–**i**) the particles in overfocus after irradiation with 10 keV He, 10 keV He + 5 keV D and 10 keV He + 5 keV D + 1.7 MeV Au, respectively. (**a**,**c**,**d**,**f**) were taken at −518-nm defocus, and (**b**,**e**) were taken at −1-μm defocus. (**g**,**i**) were taken at +518-nm defocus, and (**h**) was taken at +1-μm defocus. The electron beam was on during the single and triple ion irradiations, but off during the majority of the double ion irradiation. The dashed circle in (**e**) indicates the region where cavities are most visible. All TEM images were taken at the same magnification.

Initial and final states of LiAlO_2_ powders irradiated in situ at 310 °C for 120 min are shown in Figure 12. LiAlO_2_ powders will form small voids due to 200 keV electron beam irradiation alone, likely due to displacement of Li or O atoms by the electron beam. The electron beam-induced void production rate varies depending on the particle, resulting in very complicated in situ void analysis. Voids have been observed to form due to the electron beam alone after as little as 1–2 min of exposure. All cavities in Figure 12 where confirmed by through-focal imaging. In the single and triple ion irradiation experiments, the electron beam was left on, while in the dual ion experiment, the electron beam was left off, except for imaging and alignments. In the single ion He implantation experiment (Figure 12d,g), several cavities were observed at the edges of the particle. After the double ion experiment, He + D (Figure 12e,h), small bubbles were observed at the edges of the particle (thinnest regions with less contrast) after an hour of irradiation. Due to contrast changes throughout the irradiation, the exact time of initial bubble formation was not easily determined, but estimated to be after a fluence of 1.5 × 10^17^ ions/cm^2^ (≈16 at %) under He implantation alone and under dual ion (He + D) after 1.7 × 10^17^ He/cm^2^ (≈19 at %) and 3.4 × 10^17^ D/cm^2^ (≈20 at %). This suggests that He content may be the dominating factor in bubble nucleation, independent of the ^2^H content inside the material. Note that the gas concentrations in parentheses are the peak values calculated by SRIM and are not representative of the actual gas concentration remaining inside the sample, which is unknown due to an unknown rate of outward diffusion of He and D to surfaces.

**Figure 13:**
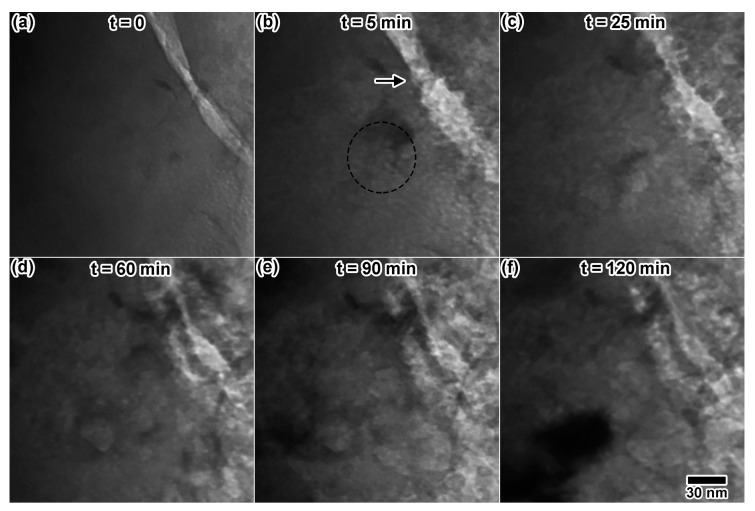
In situ TEM images showing microstructural evolution of LiAlO_2_ under triple ion irradiation with the electron beam on the sample. (**a**) The initial microstructure appears void-free, but (**b**) cavity-like structures appear after 5 min and continue to grow in (**c**,**d**) until large blisters appear to crack the microstructure in (**e**,**f**). Dashed circles indicate a region containing easily-visible defects believed to be cavities. All TEM images were taken at the same magnification.

The particle utilized for triple ion irradiation (Figure 12c) did contain some pre-existing voids, but most of the particle was void-free. Triple ion irradiation resulted in an entirely different microstructure (Figure 12f,i) than double and single ion irradiation. Though examination of the final state images indicates a drastic change in the microstructure, the mechanism for this change cannot be elucidated with final images alone. A closer look at select in situ images is provided in Figure 13. Figure 13a shows a region with very few pre-existing voids. After just 5 min of triple ion irradiation, Figure 13b shows a significant increase in the cavity density. Some possible cavities are circled in the image. Increasing the irradiation time appears to increase the void size, as shown at 25 min in Figure 13c, after 60 min in Figure 13d and after 90 min in Figure 13e. The final microstructure (Figure 13f) appears to contain large blisters that possibly caused cracking of the material. Drastic changes in the crack microstructure, indicated after 5 min by an arrow in Figure 13b, can also be observed throughout the in situ irradiation. The final microstructure could not be focused to a single eucentric point inside the microscope, indicating a significant increase in topology. Some of this microstructural change is likely due to synergy between ion irradiation and the electron beam, so other particles were imaged after the irradiation. Large, platelet-like cavities were observed in other particles, with less blistering and cracking of the microstructure. It is difficult to distinguish between areas that were exposed to the electron beam and areas that were not exposed, however.

## 4. Discussion

The He + D beam emitted from the Colutron is an interesting study in ion accelerator physics. A full characterization of this source is beyond the scope of this paper; however, it has been noted that the beam current varies with the partial pressures of the two source gasses in a manner that does not appear to be completely linear. Experiments in this work utilized the same accelerator settings for consistency. As stated earlier, the ERD experiments consistently revealed fluences approximately one order of magnitude lower than those estimated by the Faraday cup current and also consistently found a D to He ratio of approximately 1.8. Lower measured He and D concentration is probably due to some gas diffusing to and escaping at the surface between the time of implantation and the time of the ERD measurement. Fluences calculated using the Faraday cup current should be of reasonable accuracy and have thus been used throughout the paper for the reported fluences of different ion species.

Systematic experiments on Au, a model face-centered cubic (FCC) crystal structure, were utilized to gain a basic understanding of the type of synergistic effects one might see during an in situ triple ion irradiation experiment, compared to in situ single and double ion irradiation. The results here suggest that ion flux may have some effect on the final structure of the defects. It should be noted that the grains in the low and high flux cases presented here were not oriented identically with respect to the electron beam, so a quantitative comparison is unwarranted. However, a qualitative reason for this observation is that defect clusters produced by MeV-range Au ions in Au often evolve over seconds or minutes. In the low flux experiment, defect clusters often remained undisturbed for seconds to tens of seconds (if not longer) before interacting with another cascade produced by the next incoming ion. The case was quite different with the high flux case, where cascades rapidly occurred one after another. The effects of flux in in situ ion irradiation TEM experiments remain an intriguing, but incompletely explored area. Dose rate effects of heavy ion irradiation were first explored to help refine the experimental parameters for triple ion irradiation experiments. In situ ion irradiation allows for observation of individual damage events and quantification of damage formation as a function of time. In a larger grained material, the two samples could be oriented to the same diffraction condition to quantify the changes in defect density, and therefore defect recovery properties, as a function of ion flux.

In situ single ion irradiations illustrated the most fundamental defects one would expect to occur due to the individual ions, with the same ion flux and fluence, without any synergistic effects. Due to the lower mass and energy of He and D in these irradiations, only small black spots resulting from point defect production were observed. In comparison, the heavy ion, higher energy Au irradiation produced displacement cascades that resulted in a visibly more complex defect structure, even though the Au ion flux was three orders of magnitude lower than the He and D flux. Of all single ion irradiation experiments, cavities were only observed in the case of He ion irradiation. Combining ion beams with in situ double ion irradiation resulted in different microstructures. In the sample implanted with He + D, no bubble formation occurred, only small black spot formation, after 30 min. This, combined with the lack of bubble formation in single ion D-irradiated Au, indicates that bubble nucleation may be dominated by He in Au; after 30 min, the He concentration in the He + D experiment was estimated to be half of that achieved during the single ion irradiation experiment. If bubble nucleation were equally influenced by both gas species, bubbles should be observed at the same fluence in both He and He + D irradiations. Though no cavities were observed in samples irradiated with the Au beam alone, dual ion irradiation with D + Au and He + Au, to approximately the same Au fluence, resulted in cavity formation in both samples. This result compliments the results of Chisholm et al., where successive Au irradiation followed by He implantation and He implantation followed by Au irradiation resulted in no cavity formation, but in situ dual beam irradiation with He + Au did result in cavity formation [23], suggesting a propensity for cavity formation in Au under dual ion irradiation. During multi-ion irradiation, gas atoms may act to stabilize voids produced by displacement cascades, eventually resulting in visible cavities. Cavities did seem to form more readily in samples irradiated He + Au and D + Au than in samples irradiated with He + D. Cavity-like structures were observed after 10 min in the sample irradiated with He + Au, a much lower He concentration than the sample irradiated with He + D, where cavities were not observed, after 30 min of irradiation. This could be due to the expected decrease in He mobility as the microstructure evolves [24]. Significantly more microstructural evolution occurs during Au irradiation than during implantation with He, D, or both simultaneously. In addition, larger vacancy clusters may form due to cascade damage than the point defect damage produced by He or D irradiation, creating additional trapping sites for He atoms, allowing them to more readily nucleate cavities. Nevertheless, the combination of displacement cascade damage and gas accumulation seems to create a more favorable environment for cavity formation than gas implantation alone. These cascade-gas interactions seem to be the dominant synergistic effect in Au, as evidenced by the significant difference in cavity growth between single and dual ion irradiations and the lack of a significant difference between dual and triple ion irradiations. Cavity formation and disappearance, shown in Figure 9, were observed in a few instances during both double ion D + Au irradiation, as well as during triple ion irradiation. Displacement cascade damage has been shown to influence He bubbles in materials. Donnelly et al. [25] saw athermal migration, coalescence and disintegration of He bubbles in Au under 400 keV Ar ion irradiation at 227 °C. They formed underpressurized bubbles in Au by He ion irradiation and subsequent annealing, but Ar irradiation caused the bubbles to reach equilibrium pressure after only 0.6 dpa. Bubble disappearance is thought to be due to either bubble migration to the surface, followed by crater formation and surface reconstruction, or by a single cascade event resulting in He re-solution. Cascade-induced He re-solution has also been hypothesized by Ghoniem [24]. 

In zirconium alloys, single ion He implantation, double ion He + Zr irradiation and triple ion irradiation with He, D and Zr at 310 °C resulted in similar microstructures. These samples seemed to form defects that were much more difficult to study, presumably due to the complex chemistry and propensity for hydride formation in ZIRLO and Zircaloy. No bubbles were observed in situ, even after irradiating at 310 °C overnight, but cavities were observed in the same samples 30 days post-irradiation. The reasoning for bubble formation post-irradiation is not well understood, as He should be mobile at room temperature in zirconium [26]. Speculating, this behavior could be due to electron beam effects or complex He-V or He-D-V cluster diffusion processes may be leading to bubble formation on a longer timescale. In LiAlO_2_ samples irradiated in situ at 310 °C with He, He + D and He + D + Au irradiation resulted in significantly different microstructures. In this material, deconvoluting electron beam effects is difficult, as the electron beam has been observed to produce small voids at different rates in different particles. Additionally, many particles have a pre-existing nano-sized void concentration. Interestingly, the He concentration required for bubble formation in the single and double ion irradiation experiments was similar, indicating that He dominates bubble formation in this material and that the D concentration does not have an effect on He bubble nucleation. Similar behavior was observed in the Au ion irradiation experiments. Triple ion irradiation resulted in a significantly different microstructure than single and dual ion irradiation. Single and dual ion irradiation experiments resulted in a low density of nanometer-scale bubbles. Examination of in situ images from the triple ion irradiation experiment elucidated some of the irradiation damage process. Though the sample did contain some pre-existing voids, several regions within the observable area were free of voids. Cavity-like structures appeared after 5 m and seemed to grow into larger, platelet-like structures with time. By the end of the 2-h irradiation, all structures previously believed to be cavities had evolved into large, platelet-like structures, possibly blisters, which appeared to crack and break apart the microstructure. 

## 5. Conclusions

This novel technique of in situ triple ion beam irradiation provides an excellent method for deconvoluting reactor and other complex irradiation environments. When done sequentially with single and double ion irradiation, these experiments elucidate kinetic processes previously difficult to understand through experimental efforts. While real-time observation of defect evolution due to ion irradiation is only available through this technique, electron beam effects, such as electron beam-induced void formation, and thin film effects, such as defect and gas migration to free surfaces, influence the microstructural evolution and must be considered. This initial work illustrates the type of synergistic interactions observable by combining different irradiation conditions and provides a foundation for understanding the fundamental interactions of defects in different materials and environments.

## References

[1] Zinkle S.J., Was G.S. (2013). Materials challenges in nuclear energy. Acta Mater..

[2] Beck L., Serruys Y., Miro S., Trocellier P., Bordas E., Leprêtre F., Brimbal D., Loussouarn T., Martin H., Vaubaillon S. (2015). Ion irradiation and radiation effect characterization at the JANNUS-Saclay triple beam facility. J. Mater. Res..

[3] Kurashima S., Satoh T., Saitoh Y., Yokota W. (2017). Irradiation facilities of the Takasaki Advanced Radiation Research Institute. Quantum Beam Sci..

[4] Tanaka T., Oka K., Ohnuki S., Yamashita S., Suda T., Watanabe S., Wakai E. (2004). Synergistic effect of helium and hydrogen for defect evolution under multi-ion irradiation of Fe-Cr ferritic alloys. J. Nucl. Mater..

[5] Butler E.P. (1979). In situ experiments in the transmission electron microscope. Rep. Prog. Phys..

[6] Ishino S. (1997). A review of in situ observation of defect production with energetic heavy ions. J. Nucl. Mater..

[7] Birtcher R.C., Kirk M.A., Furuya K., Lumpkin G.R. (2017). In situ transmission electron microscopy investigation of radiation effects. J. Mater. Res..

[8] Hinks J.A. (2009). A review of transmission electron microscopes with in situ ion irradiation. Nucl. Instrum. Methods Phys. Res. Sect. B.

[9] Kirk M.A., Baldo P.M., Liu A.C.Y., Ryan E.A., Birtcher R.C., Yao Z., Xu S.E.N., Jenkins M.L., Hernandez-mayoral M., Kaoumi D. (2009). In situ transmission electron microscopy and ion irradiation of ferritic materials. Microsc. Res. Tech..

[10] Pashley D.W., Presland A.E.B. (1961). Ion damage to metal films inside an electron microscope. Philos. Mag..

[11] Howe L.M., McGurn J.F., Gilbert R.W. (1966). Direct observation of radiation damage produced in copper, gold, and aluminum during ion bombardments at low temperatures in the electron microscope. Acta Metall..

[12] Whitmell D.S., Kennedy W.A.D., Mazey D.J., Nelson R.S., Kennedy W.A.D., Mazey D.J., Nelson R.S.A. (2017). A heavy-ion accelerator-electron microscope link for the direct observation of ion irradiation effects. Radiat. Eff. Defects Solids.

[13] Hattar K., Bufford D.C., Buller D.L. (2014). Concurrent in situ ion irradiation transmission electron microscope. Nucl. Instrum. Methods Phys. Res. Sect. B Beam Interac. Mater. Atoms.

[14] Nastasi M., Mayer J.W., Wang Y.-Q. (2014). Ion Beam Analysis: Fundamentals and Applications.

[15] Rasband W.S. ImageJ, US National Institutes of Health. http://imagej.nih.gov/ij/.

[16] Ziegler J.F., Biersack J.P., Littmark U. (1985). The Stopping Range of Ions in Solids.

[17] Broeders C.H.M., Konobeyev A.Y. (2006). Development of Calculation Methods to Analyze Radiation Damage, Nuclide Production and Energy Deposition in ADS Materials and Nuclear Data Evaluation.

[18] Lizunov Y., Möslang A., Ryazanov A., Vladimirov P. (2002). New evaluation of displacement damage and gas production for breeder ceramics under IFMIF, fusion and fission neutron irradiation. J. Nucl. Mater..

[19] Edington J.W. (1976). Practical Electron Microscopy in Materials Science.

[20] Donnelly S., Birtcher R. (1997). Heavy ion cratering of gold. Phys. Rev. B.

[21] Birtcher R.C., Donnelly S.E., Schlutig S. (2000). Nanoparticle ejection from Au induced by single Xe ion impacts. Phys. Rev. Lett..

[22] Burns K.A., Love E.F., Thornhill C.K. (2012). Description of the Tritium-Producing Burnable Absorber Rod for the Commercial Light Water Reactor.

[23] Chisholm C., Hattar K., Minor A.M. (2014). In Situ TEM concurrent and successive Au self-ion irradiation and He implantation. Mater. Trans..

[24] Ghoniem N.M. (1990). Nucleation and growth theory of cavity evolution under conditions of cascade damage and high helium generation. J. Nucl. Mater..

[25] Donnelly S.E., Birtcher R.C., Templier C., Vishnyakov V. (1995). Response of helium bubbles in gold to displacement-cascade damage. Phys. Rev. B.

[26] Lewis M.B., Farrell K. (1986). Migration behavior of helium under displacive irradiation in stainless steel, nickel, iron and zirconium. Nucl. Instrum. Methods Phys. Res. Sect. B.

